# Implementation of a motion estimation algorithm for Intel FPGAs using OpenCL

**DOI:** 10.1007/s11227-023-05051-3

**Published:** 2023-01-21

**Authors:** Manuel de Castro, Roberto R. Osorio, David L. Vilariño, Arturo Gonzalez-Escribano, Diego R. Llanos

**Affiliations:** 1 Departamento de Informática, Universidad de Valladolid, Escuela de Ingeniería Informática, Campus Miguel Delibes, Paseo Belén 15, 47011 Valladolid, Spain; 2 CITIC, Computer Architecture Group, Universidade da Coruña, Campus de Eviña s/n, 15008 A Coruña, Spain; 3 Departamento de Electrónica y Computación, Universidad de Santiago de Compostela, Campus Vida s/n, 15782 Santiago de Compostela, Spain

**Keywords:** FPGA, OpenCL, Motion estimation, Video coding

## Abstract

Motion Estimation is one of the main tasks behind any video encoder. It is a computationally costly task; therefore, it is usually delegated to specific or reconfigurable hardware, such as FPGAs. Over the years, multiple FPGA implementations have been developed, mainly using hardware description languages such as Verilog or VHDL. Since programming using hardware description languages is a complex task, it is desirable to use higher-level languages to develop FPGA applications.The aim of this work is to evaluate OpenCL, in terms of expressiveness, as a tool for developing this kind of FPGA applications. To do so, we present and evaluate a parallel implementation of the Block Matching Motion Estimation process using OpenCL for Intel FPGAs, usable and tested on an Intel Stratix 10 FPGA. The implementation efficiently processes Full HD frames completely inside the FPGA. In this work, we show the resource utilization when synthesizing the code on an Intel Stratix 10 FPGA, as well as a performance comparison with multiple CPU implementations with varying levels of optimization and vectorization capabilities. We also compare the proposed OpenCL implementation, in terms of resource utilization and performance, with estimations obtained from an equivalent VHDL implementation.

## Introduction

In recent years, there has been an increment in the generation and consumption of video-based media, due to the popularity of video streaming services such as Netflix, YouTube and HBO, and to the increment in usage of teleconferencing platforms as a consequence of the COVID-19 pandemic. Video, as a digital media, contains huge amounts of information, making its uncompressed usage prohibitive. It is due to video compression that the multimedia revolution we are experiencing is possible in the first place.

Advanced video encoders make use of Motion Estimation and Compensation algorithms to achieve high compression rates. Motion is commonly estimated using a Block Matching technique, which divides a given frame image into blocks of pixels, and tries to find the closest match for each block within one or more previously encoded frames. In this way, large blocks of pixels may be encoded as a motion vector, a spatial reference to a matching block.

Block Matching is responsible for the highest compression gains in video coding. It is a computing-intensive task, but it is also desirable to be performed in real time in a multitude of scenarios. Due to the embedded or low-consumption nature of most of the devices that perform video coding tasks, hardware solutions are more popular than programmable ones. Thus, Block Matching is usually implemented by means of application specific hardware, including Application Specific Integrated Circuits (ASICs), Field Programmable Gate Arrays (FPGAs), dedicated hardware in Graphic Computing Units (GPUs), and multimedia coprocessors in General Purpose Processors (GPPs).

A large number of techniques and heuristics have been proposed to reduce the computational load of Block Matching. Hence, modern implementations try to minimize the sum of absolute differences between pixels; although the more costly (and more accurate) sum of squared differences was originally proposed. In addition, the Full Search algorithm [1] gives the best results, but heuristics such as Diamond Search [2] greatly reduce the number of computations. Motion vectors can also be predicted, narrowing the search space. Finally, some papers propose comparing the value of only some pixels in the blocks, or even averaged values. All these techniques reduce the number of computations at the expense of reducing accuracy. As an additional step, modern video standards implement fractional Motion Estimation in order to further improve compression.

Video encoders often allow the accuracy level to be selected, so the user can prioritize either the encoding time or the compression ratio. This makes sense, as the encoder may work both in a real-time encoding scenario, or in an offline application in which content is encoded just once, but it is transmitted, stored, and reproduced many times.

FPGA devices are rising in popularity as accelerators in supercomputers since they are able to accelerate problems that other accelerators, namely SIMD ones such as GPUs, are not. Besides, FPGAs offer higher energy efficiency (i.e., performance per watt) than CPUs and GPUs for many interesting HPC applications. ASICs, for their part, might offer even higher performance and energy efficiency for specific applications; however, they lack the flexibility of FPGAs, which makes them unsuitable for general-purpose computing. Nevertheless, FPGAs are often programmed using Hardware Description Languages (HDLs), such as VHDL and Verilog. These languages have high development costs, specially for software programmers, due to their low-level scope. To ease these costs, High Level Synthesis (HLS) design environments have been developed, such as Xilinx HLS [3], and SystemC [4]. These leverage high-level programming languages, generally C-based ones, to abstract most of the low-level details and bring FPGA programming closer to software programming.

OpenCL [5] has also been adapted to work as an HLS environment targeting FPGA devices. As a framework, it uses a C-based high level language to program hardware accelerators. It is a choice of special interest, given the widespread adoption of the framework in the HPC community to develop applications targeting heterogeneous systems, especially those using GPUs. OpenCL’s main focus is to enable code portability among different kinds of heterogeneous devices. Thus, unlike other HLS environments, OpenCL can be used to develop programs that target at the same time CPUs, GPUs, FPGAs, and other kinds of accelerators. Moreover, as OpenCL is already a well-known language among many members of the HPC community, its adoption for developing HPC applications targeting FPGAs should be more straightforward than using other HLS environments.

With the increasing popularity of heterogeneous systems in media centers comprising, among others, high-end data center FPGAs, we consider OpenCL a promising choice for implementing high-efficiency video processing applications. To the best of our knowledge, there is not any previous implementation of Motion Estimation on FPGAs using OpenCL. More specifically, Intel offers implementations targeting GPUs, but none for FPGAs. This is somewhat surprising, coming from a company that is both an FPGA manufacturer and an earlier supporter of OpenCL.

While it is commonly assumed that leveraging accelerators using OpenCL should bring clear benefits to the task in terms of speed-up and/or power consumption, this is not always the case. Particularly, FPGAs have two very important disadvantages compared to GPCPUs and GPGPUs: Lower clock speed and larger power consumption per computation. Engineers are able to overcome or partially compensate these disadvantages by exploiting the main strengths of FPGAs: Fine and coarse grain parallelism, and low overhead in computations. It is well known that Block Matching exhibits high parallelism, which FPGAs are able to exploit. In this work, we demonstrate that OpenCL is well suited to detect and exploit the existing parallelism. On top of that, OpenCL offers flexibility and ease of programming, which is crucial to achieve the level of productivity required for the development of modern systems.

In our research, we target the acceleration of Motion Estimation on FPGAs. With the large number of possible implementations, we plan to reduce the development cost by using OpenCL. In this paper, we have tackled the most straightforward implementation, Full Search, as well as a preliminary implementation of Diamond Search. We have assessed the capabilities of OpenCL to describe and synthesize fully parallel architectures.

The main goal of this work is to test the expressiveness of OpenCL as a design language for Block Matching Motion Estimation and other similar applications, assessing the quality of the implementation and comparing it to hand-optimized ones. To contribute to open science, the source codes and compilation reports generated during the development of this work are freely available on the following repository: https://gitlab.com/mandeca/me_opencl.

The rest of the paper is organized as follows. Section 2 discusses related work on the field of Motion Estimation implementations for FPGAs; Sect. 3 describes the Block Matching Motion Estimation process; Sect. 4 describes the framework used to develop the solution (OpenCL for Intel FPGAs); Sect. 5 details the development process of our proposal, and the features of the different versions developed; Sect. 6 evaluates our proposals and OpenCL as a tool for developing video processing applications targeting FPGAs; and lastly, sect. 7 discusses the conclusions and future work.

## Related work

Block Matching Motion Estimation is the cornerstone in most advanced video encoders. It is a task that involves high amounts of computation, even when the search of similar blocks is restricted to the closest vicinity. It is the most expensive task in video coding, in terms of computation time.

For this reason, research works have sought for fast and efficient architectures to accomplish this task [6]. Beyond the basic Full Search algorithm, several alternatives have been proposed [7–9] that achieve great computational savings at the cost of only a small loss in accuracy. Also, architectures able to deal with variable block sizes [10] and fractional pixel interpolation [11] allow data compression to be maximized.

The use of FPGAs as accelerators in video encoding is of great interest. FPGA-specific implementations take advantage of the availability of embedded memories, which allow for fast access to internally cached data [12, 13].

The advent of high-level hardware synthesis languages, such as OpenCL, opens a new era in the implementation of custom architectures. Previous work discussing OpenCL as a framework for developing FPGA applications and its benefits includes [14–16].

While some works have been published about implementing Block Matching using OpenCL, proving its validity to accelerate the task, the targeted platforms are either CPUs or GPUs [17–20], as FPGAs are still programmed using HDLs or other HLSs. To the best of our knowledge, no OpenCL implementation that also targets FPGAs has been previously published, and Xilinx HLS is the highest level language for which Block Matching architectures have been published.

In [21], an architecture for Motion Estimation that is not FPGA oriented is proposed, and implements Diamond Search. In [22], an FPGA-based programmable processor with multiple processing units oriented to H.264 video coding is proposed. They both have in common that parallel processing is achieved by means of multiple parallel memories.

Finally, in [23] a highly parallel architecture for Motion Estimation in H.265 is proposed. There are some interesting similarities with our work. First, data access is achieved by implementing sets of 64 parallely-accessed memory blocks, as many as pixels in an $8 \times 8$ block. Second, results for a FPGA implementation are given that concur with the ones obtained in our work. Chiefly, the combined area of Motion Vector generator and cost estimator is less than 34% of the available LUTs (in an old Arria II device), which is significantly less than the 52% required by the interpolator. Therefore, as in our work, implementing a highly parallel architecture for Motion Estimation is the way to achieve high processing speed, and it is not limited by resource utilization. The speed in the referenced paper is limited to 200 MHz but, again, this is due to using an old device. However, this work is different from our contribution in many important aspects. First, unlike our work, the referenced paper is not restricted to full-pixel Motion Estimation, but also implements fractional-pixel Motion Estimation by means of interpolation. Second, full-pixel Motion Estimation is computed for $8 \times 8$ pixel blocks, and Sum of Absolute Differences results for $16 \times 16$ and larger blocks are obtained by adding up results for $8 \times 8$ blocks. In our work, Motion Estimation is computed for $16 \times 16$ blocks directly. Finally, it has been implemented using VHDL, instead of a high level language as we do.

## Block matching motion estimation

Motion Estimation (ME) is a process by which the motion vectors that describe changes among different video frames are determined. In video coding, Block Matching Motion Estimation is used to compress video files by reducing temporal redundancies, and has been used since the inception of video encoders. It is the main component of inter-frame prediction, and provides the highest compression gains in any video standard, such as AVC or HEVC. Nevertheless, it is also the most computationally costly task performed in video coding, corresponding to more than half the computation time of the whole process.

ME divides the current encoding frame into non-overlapped small blocks, called *macroblocks*. The size of the macroblocks can be variable and irregular, but $16\times 16$ is a usual choice in classic encoders. ME is applied over all the macroblocks in the frame, attempting to find for each one of them the most similar macroblock among a set of candidates from a reference frame (a previously encoded frame). Thus, blocks of pixels can be represented in the encoded video as a motion vector representing the movement of the similar macroblock, the index of the reference frame in which the similar macroblock is found, and the prediction error to reconstruct the original macroblock.

**Fig. 1:**
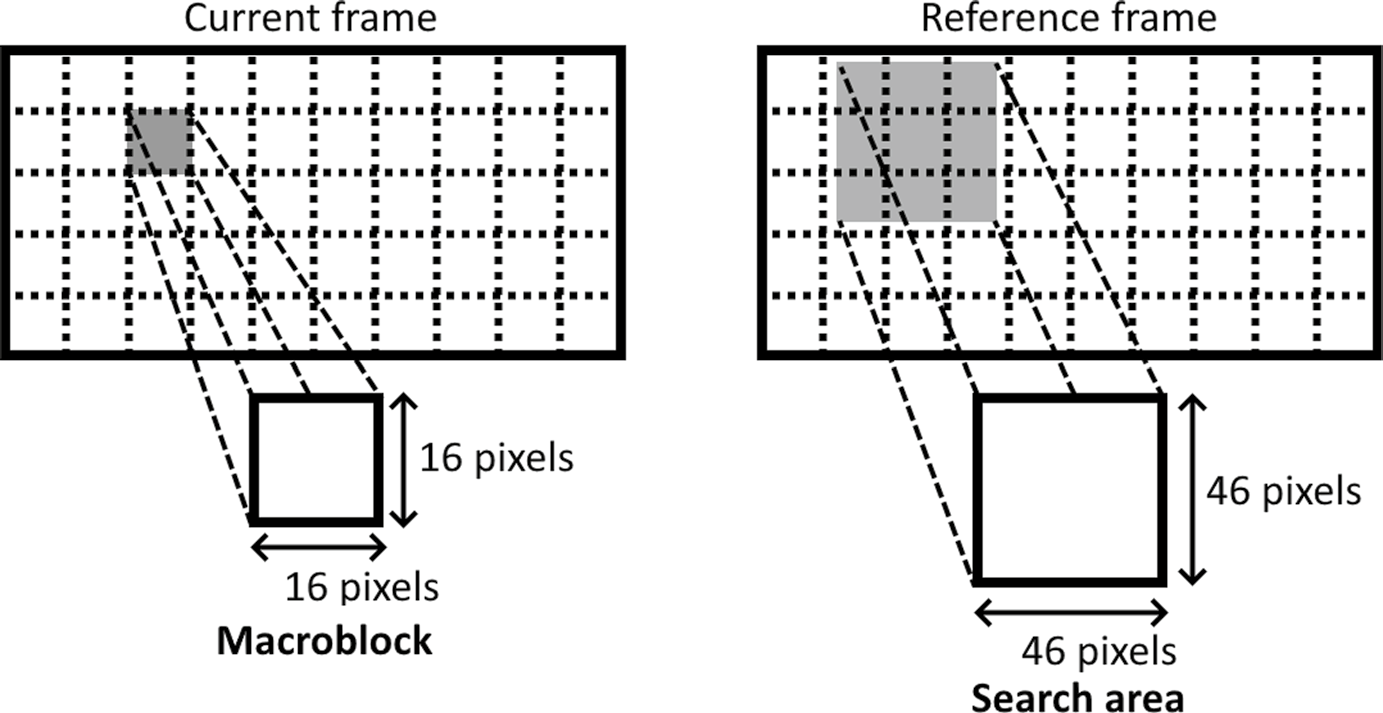
Division of a frame into macroblocks, and search area corresponding to a given macroblock

The set of candidate macroblocks to be checked is determined by a search area and a search method. The search area is usually restricted to the vicinity of the relative position of the macroblock, but in the reference frame. Figure 1 shows the subdivision of frames into macroblocks, and an example of the search area for a given macroblock. Inside the search area, the candidate macroblocks are overlapped, i.e., any block of pixels inside the search area that has the same dimensions as the macroblock to be encoded is a valid candidate.

To determine the most similar macroblock among the candidates, similarity criteria are used, such as Sum of Absolute Differences (SAD) and Sum of Squared Error (SSE), between the macroblock to be encoded and the candidates. SAD is the most commonly used option in video encoders due to its low computational complexity, even though SSE is more accurate. It is computed as shown in Eq. 1, where *w* and *h* are the width and height of the macroblock, respectively, *Ref* is the candidate macroblock, and *Cur* is the current macroblock.

1 $$\begin{aligned} SAD = \sum _{j=0}^{w-1}\sum _{i=0}^{h-1} |Ref_{i,j} - Act_{i,j} | \end{aligned}$$

**Fig. 2:**
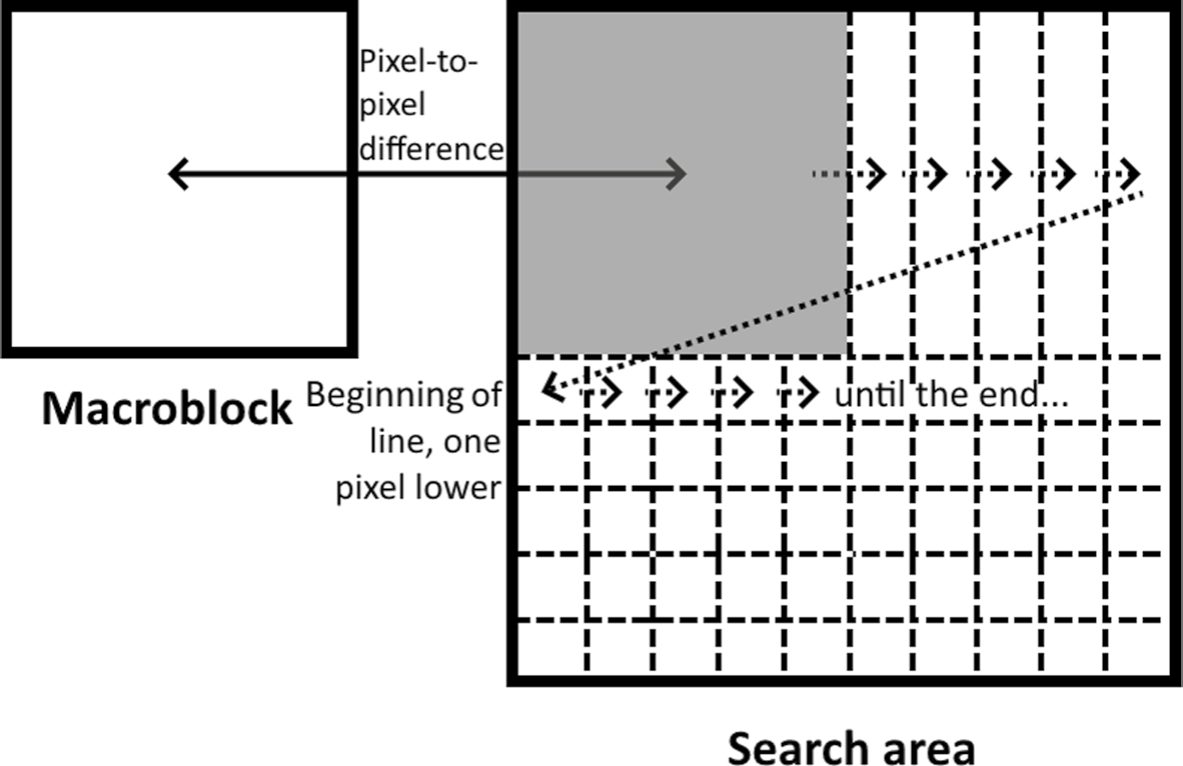
Computation of the similarity between a macroblock and all the candidates in a search area. The similarity value is the sum of the absolute values of the 256 computed subtractions (the lower, the more similar)

As can be seen, the SAD for a candidate macroblock is computed from multiple independent subtractions. Given the high amount of candidate blocks that can be processed per macroblock, and the high amount of macroblocks to process per frame, ME offers great optimization opportunities through parallel implementations. At the same time, while the computation of each candidate might be independent too, the accessed data are partially overlapped, allowing for techniques that exploit locality to increase throughput.

Concerning the search method used to determine which candidates in the search area should be computed, there are multiple choices. Full Search [1] computes every possible candidate, always finding the optimal solution. Figure 2 illustrates how Full Search could be performed for a given macroblock and its corresponding search area. However, Full Search is not a viable option in real-time encoding scenarios. Other methods use heuristics to reduce considerably the amount of candidates computed to accelerate the task, allowing for suboptimal solutions. These include, among others, Three Step Search [24], New Three Step Search [25], Four Step Search [26], Block-Based Gradient Descent Search [27], Diamond Search [9], Hexagon Search [28], and Test Zone Search [29].

## OpenCL for Intel FPGAs

Our proposed implementation is developed using OpenCL for Intel FPGAs. This section introduces OpenCL and the OpenCL for Intel FPGAs framework.

### OpenCL standard

OpenCL [5] is an open standard for parallel programming in heterogeneous systems. Its main aim is to allow the user to write portable parallel programs among different kinds of computing systems such as CPUs, GPUs, and other accelerators, with minimal-to-no changes in the source code targeting different systems. Thus, all available resources in the system can be exploited to achieve high performance.

**Fig. 3:**
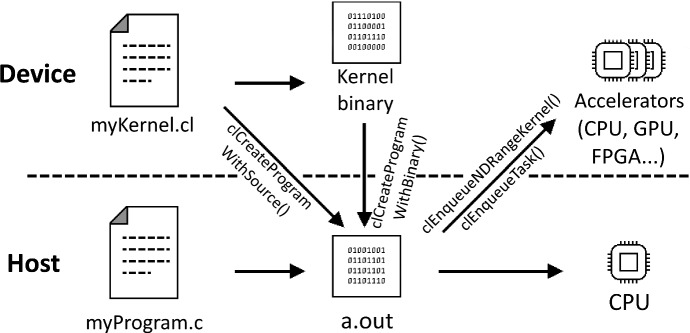
Different executable components of an OpenCL application and their interactions

The OpenCL programming model differentiates between host and devices, both at an application level and at a system resources level. The host is the CPU that executes the main part of the program and coordinates the devices, whereas the devices are the computational units of the systems, such as CPUs, GPUs, FPGAs, or other accelerators, which are intended to execute and accelerate computationally-intensive parts of the program. Device-executed code is written as kernel functions using OpenCL C language (a C99 dialect). The host code is commonly written in C or C++, although it can also be written in other languages such as Python. Figure 3 shows the interactions between host and devices during a common OpenCL program execution.

### OpenCL for Intel FPGAs

Included in Intel’s oneAPI suite is the Intel FPGA SDK for OpenCL (which we refer to in this work as “OpenCL for FPGAs”). This framework allows the compilation of OpenCL kernels into Intel FPGA bitstreams, being compliant with the OpenCL 1.0 standard. Since the compilation times for FPGA applications are very high, OpenCL for Intel FPGAs only allows for the offline compilation of kernels (i.e., not in execution time, as regular OpenCL programs usually do). To ease the debugging process, it also provides FPGA emulation capabilities. Thus, an emulation kernel can be compiled in seconds and executed on CPU to check its correctness.

During the compilation process of an Intel FPGA kernel, the compiler generates a compilation report. This contains useful information regarding the FPGA bitstream being generated, such as the working frequency of the design, the amount of FPGA resources used, loop performance analysis, memory hierarchy usage, and design schematics.

OpenCL for Intel FPGAs allows fine-tuning of the kernels via preprocessor directives (C pragmas) and compilation flags. The user can control, for example, how the kernel’s loops are pipelined or unrolled, in which type of memory the arrays should be allocated, or the amount of vector lanes to use.

## Our proposal

In this section, we describe the methodology followed when developing our proposal, as well as the features of the final versions.

### Development process

We decided to develop the kernels in an incremental manner, analyzing how OpenCL implements each small feature of the algorithm before adding anything else. During such analysis, we compile the same kernel several times, tweaking certain parameters and compare the resulting compilation reports. The compilation reports generated by HLS frameworks usually present good theoretical performance evaluations, which can be used instead of more costly, experimental studies to make comparisons between different kernel versions, which are more appropriate for near-production kernel versions. Nevertheless, Intel’s reports do not provide an estimate for the design’s latency. When provided, this metric is useful to evaluate a kernel’s performance in an absolute, end-user friendly way, as it can be used alongside the reported working frequency to estimate the kernel’s execution time. In this work, the main theoretical results used to evaluate a kernel’s quality were the design’s working frequency, the memory type used to implement the buffers, the resource utilization, and the loop performance analysis.

We decided from the beginning to develop a kernel targeting the Intel Stratix 10 FPGA, a data center FPGA model. Being a data center FPGA, the high amount of resources it contains is suitable for developing complex, resource-consuming OpenCL kernels without compromising their performance. Among all the existent data center FPGA models, the Stratix 10 model was chosen because it is a state-of-the-art FPGA device. One downside choosing a data center FPGA poses is that the FPGA and proposed designs will not be applicable to low-consumption and embedded computing scenarios. In those cases, other FPGA models, including those of other vendors such as Xilinx, may be more suitable, together with smaller-footprint designs.

We used the naäve C implementation found at [30] as baseline, and decided that our proposal would work with $16\times 16$ pixel macroblocks, and $46\times 46$ pixel search areas. The first kernel we developed only performed the SAD operation on the FPGA. Being a small kernel, it allowed us to delve into all the characterization parameters that OpenCL for Intel FPGAs present, in order to tweak the kernels. The second kernel we developed processed individual macroblocks, with all their 961 Full Search candidates, on the FPGA. The design that Intel’s compiler returned for both kernels seemed reasonable enough, resembling what a low-level manual implementation could look like (with additional circuitry for internal OpenCL logic). After this, the development of a full-frame-processing kernel started.

### Final full search versions

Two Block Matching implementations using Full Search have been implemented as OpenCL kernels targeting Intel FPGAs. Both versions work with Full HD video frames ($1\,920\times 1\,080$ pixels), a luminance component only (as it is often done in other works in the field), fully inside the FPGA. The macroblock size is $16\times 16$ pixels, and the search area is $46\times 46$ pixels, for a total of 961 candidate macroblocks computed per macroblock. The frames are extended 8 pixels in height by duplicating the pixels on the last line 8 times, to allow for an exact division of the frame in macroblocks. Each frame contains exactly $8\,160$ macroblocks, which results in $7\,344\,000$ motion vectors computed per frame.

Both kernels receive, as parameters, pointers to the current and reference frames, and three pointers to store the kernel results: The minimum SAD found for each macroblock, the *x* component of the motion vectors corresponding to the minimum SAD, and the *y* component of the motion vectors corresponding to the minimum SAD.

All buffers of pixels, including current and reference frames, macroblock and search area, are represented using one-dimensional arrays of bytes. Each pixel is represented by a single unsigned byte, which encodes the brightness of the pixel; i.e., 0 represents a fully black pixel, whereas 255 represents a fully white pixel. Color information is discarded, as it is not very useful for performing Motion Estimation. The bigger buffers, corresponding to both frames, are stored in global FPGA memory (DDR4), and the smaller buffers, macroblock and search area, are stored in faster internal FPGA memory.

Our proposal is able to achieve a high acceleration by exploiting two main techniques which complement each other:

- Memory hierarchy exploitation. Local OpenCL memory, which is synthesized as internal FPGA memory, presents a data throughput which is orders of magnitude faster than global memory. By preloading highly reused data in local memory before the computation, the performance of our proposal is considerably increased. Specifically, the current macroblock and search area are allocated in internal memory. The current macroblock, which is 256 bytes in size, is implemented using 128 MLAB registers. The current search area, which is $2\,116$ bytes in size, is implemented using 2 internal M10K SRAMs. However, this buffer is replicated 256 times to allow 256 concurrent accesses, which results in a total usage of 512 M10Ks to allocate the current search area.
- Sum of Absolute Differences parallelization. The SAD operation, which is the computational cornerstone of Block Matching Motion Estimation, consists of multiple independent operations: as many as pixels for the chosen macroblock size. In our implementation, the amount of operations is 256, although this number can vary, usually between 16 and 4096. By unrolling the main loop of the SAD operation, these computations can be parallelized. The degree of parallelization is dependent on the amount of resources available in the FPGA, as well as memory throughput. In the case of our target FPGA, the amount of resources is more than enough to fully parallelize the 256 operations.

By exploiting both techniques simultaneously, our proposal is able to compute 1 SAD operation per clock cycle: the internal memory feeds the computational logic 256 bytes (pixels) per clock cycle; and the computational logic computes the 256 SAD subtractions fully in parallel, in a single clock cycle. Consequently, the computation of a macroblock can be carried out in as few clock cycles as candidate macroblocks are in the corresponding search area. Thus, the bottleneck for the implementation shifts from the computation to the preloading of the data in the internal FPGA memory buffers (macroblock and search area).

Our kernels are highly parallel, thus requiring a relatively high amount of FPGA resources to synthesize all the parallel computation logic. The computation logic is replicated as many times as needed to achieve the specified degree of parallelism. Our target FPGA, the Intel Stratix 10, is a data center FPGA containing a high amount of resources, so that we could design our kernels without worrying about resource limitations. When targeting smaller FPGAs, it is advisable to reduce the degree of parallelism to adapt the kernel to the reduced amount of resources, at the expense of higher computation latencies.

**Fig. 4:**
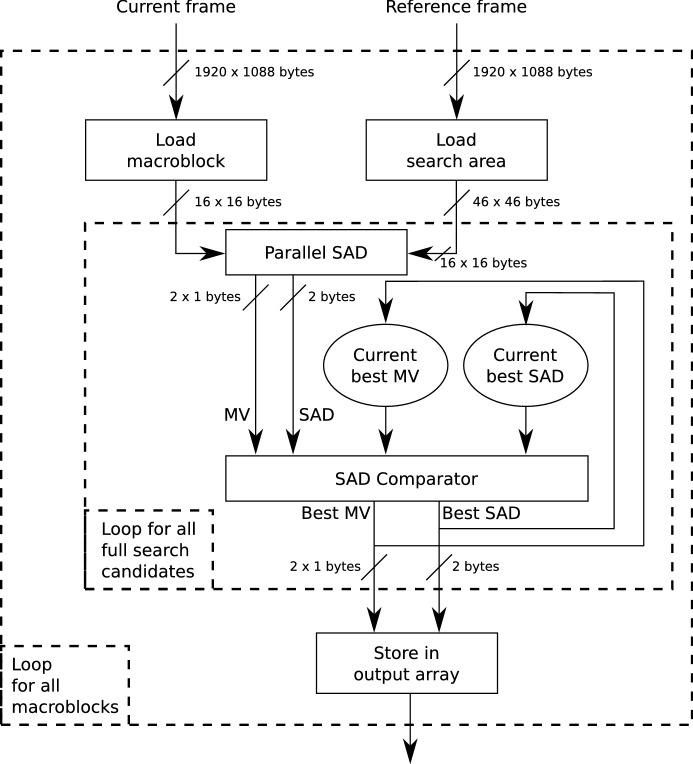
Dataflow of the developed full search block matching motion estimation kernels

The kernel dataflow is as follows: (1) The current macroblock is loaded from the current frame, residing in global memory, to internal MLAB registers; (2) the corresponding search area is loaded from the reference frame, residing in global memory, to internal M10K SRAM; (3) for each of the 961 candidates in the search area, the SAD is computed, and the minimum SAD and corresponding motion vector is stored in the result buffers; and (4) the next iteration begins, processing the next macroblock. This dataflow is depicted in Fig. 4.

The two versions developed differ in the way they handle border and corner macroblocks. These macroblocks pose additional problems as their corresponding search areas cannot be of regular size due to a lack of pixels in some of the directions. Our kernels deal with that problem as follows:

- The first kernel adds logic to detect border macroblocks and adjust the search area accordingly, computing only the valid candidates. This version computes fewer candidates per frame on average.
- The second version works with extended frames, 15 pixels in each border, so that all the macroblocks have a complete search area to work with. This resembles the way certain video encoders, such as AVC, work. This version presents less complex logic.

### Preliminary diamond search version

The high amount of computation needed to perform a Full Search makes it prohibitive for real-time encoding scenarios without heavy hardware acceleration. Most encoders use other search methods, based on heuristics, to reduce the amount of candidate macroblocks computed. One such method is Diamond Search [2]. We have developed a preliminary version of a Block Matching kernel using Diamond Search to test the suitability of OpenCL for developing real-time video processing applications for FPGAs.

The preliminary Diamond Search version developed is similar to the Full Search version that works with extended frames, only modifying the search method to use the algorithm described in [9]. Although the amount of candidates checked per macroblock decreases by approximately a factor of 30 with respect to the Full Search, this preliminary implementation did not meet our performance expectations. Section 6.2 further discusses these issues.

## Evaluation

In this section, we evaluate the developed kernels in terms of resource utilization and performance.

To the best of our knowledge, our proposal is the first implementation of Block Matching Motion Estimation that uses OpenCL and targets FPGAs. Thus, we provide a performance comparison with an equivalent CPU implementation, executed with varying degrees of optimization in current-generation CPUs.

We also consider of much interest to evaluate OpenCL-based FPGA implementations of Block Matching Motion Estimation against optimized, low-level HDL implementations. For that purpose, we have developed a VHDL implementation of the algorithm. We provide resource utilization and performance comparisons with this HDL design. The VHDL design is compiled using Quartus Prime Standard Edition v16 when targeting Intel FPGAs, and Vivado 2020 when targeting Xilinx FPGAs.

The developed VHDL architecture targets to exploit as much parallelism as the automatically generated by OpenCL. However, it is difficult for a human engineer to create and test such a complex memory architecture. Therefore, a simpler, highly regular solution has been selected. A systolic array has been devised for storing and shifting the pixels from the current macroblock and search area. Systolic arrays have been used for many years for implementing highly parallel application specific architectures [31], and recent examples for Motion Estimation can be found in the literature [32]. However, we have not found any published paper that manages $16 \times 16$ macroblocks as we do. Nevertheless, systolic architectures are highly scalable. Therefore, the architecture described in [32] is similar to our VHDL architecture, with the consideration that the cost of a $16 \times 16$ implementation is roughly 16 times the cost of a $4 \times 4$ one.

We conclude this section by providing a brief evaluation of OpenCL as a tool for developing video processing applications targeting FPGAs.

### Resource utilization

The resource utilization for our Full Search implementations, as reported by Intel’s compiler, is shown in Tables 1 and 2, both in relative and absolute terms. The FPGA system generated by the compiler from the kernel source files comprises the following parts:

- Kernel system, which comprises the hardware designs for all the compiled OpenCL kernels, the interconnect with global memory, and the system description ROM.
- Static partition, which comprises the board interface and OpenCL API logic. This is the logic responsible for managing communications with external interfaces, such as PCIe. This logic is necessary to perform communications with the host system, and cannot be modified by the user.

The static partition uses a considerable amount of the FPGA resources. It is responsible for the overhead seen in Tables 1 and 2 when comparing the *Whole system* row with the *Kernel system* row. The third row shows the total resources dedicated to the kernel logic only. The fourth row shows the estimates for resources dedicated to the kernel logic only, which are provided by the compiler before the beginning of the bitstream generation step.

**Table 1:** Stratix 10 resource utilization for the kernel that adds logic to detect border macroblocks, as reported by Intel’s compiler (*aoc*)

	ALMs	REGs	MLABs	RAMs	DSPs
Whole system	247 311 (27%)	433 503 (12%)	783 (1%)	1 198 (10%)	3 (0%)
Kernel system	52 468.9 (6%)	149 222 (4%)	783 (1%)	767 (7%)	5 (0%)
ME kernel logic	41 116.1 (4%)	115 539 (3%)	779 (1%)	587 (5%)	5 (0%)
ME kernel logic(estimated)	24 122 (3%)	92 840 (2%)	1 294 (1%)	678 (6%)	2.5 (0%)
Available	933 120	3 732 480	93 312	11 721	5 760

**Table 2:** Stratix 10 resource utilization for the kernel that works with extended frames, as reported by Intel’s compiler (*aoc*)

	ALMs	REGs	MLABs	RAMs	DSPs
Whole system	244 913 (26%)	421 377 (11%)	990 (1%)	1 187 (10%)	0 (0%)
Kernel system	50 536 (5%)	137 020 (4%)	990 (1%)	756 (6%)	0 (0%)
ME kernel logic	39 163.3 (4%)	102 273 (3%)	986 (1%)	576 (5%)	0 (0%)
ME kernel logic (estimated)	20 922.5 (2%)	72 446 (2%)	1 440 (2%)	663 (6%)	0 (0%)
Available	933 120	3 732 480	93 312	11 721	5 760

Tables 1 and 2 show that the amount of resources used by our implementations is relatively low. In both cases, the complete system uses around a quarter of the FPGAs’ resources, whereas the kernel alone uses only around 5% of any resource. In absolute terms, the resource utilization is high, especially if it is compared to the amount of resources an embedded FPGA usually has. It also shows that, for the kernel logic, the compiler tends to overestimate the amount of MLABs and RAMs the final design will use, as well as underestimate the amount of ALMs and REGs.

To put these results into perspective, we have gathered resource utilization estimates for the developed VHDL implementation. However, the synthesis tools used to generate the estimations did not allow us to target the Stratix 10 FPGA. Among the available device choices, we chose to generate estimations for the Intel Arria 10 FPGA and the Xilinx Virtex UltraScale+ FPGA. The Arria 10 family has been chosen as it is a current-generation Intel FPGA family which targets scenarios with low-and-medium performance requirements, being the Stratix 10 family the high-end alternative targeting more demanding scenarios. The Virtex UltraScale+ family has been chosen as it is another current-generation high-end FPGA targeting data center applications, with a performance comparable to that of the Stratix 10. The resource utilization estimates for the VHDL kernel are shown in Tables 3 and 4. It is worth noting that Xilinx and Intel use different names to refer to equivalent FPGA resources. For readability and ease-of-comparison reasons, Table 4 displays the resource estimates using Intel’s resource names, and following this conversion: 2 Xilinx LUTs equal 1 Intel ALM, and 1 Xilinx FF equals 1 Intel REG.

**Table 3:** Arria 10 GX 1150 resource utilization estimates for a VHDL implementation of Block Matching Motion Estimation

Intel Arria 10	ALMs	REGs
ME kernel logic(estimated)	13 165 (3%)	25 235 (1.5%)
Available	427 200	1 708 000

**Table 4:** Virtex UltraScale+ VU13P resource utilization estimates for a VHDL implementation of Block Matching Motion Estimation. Data has been transformed to the equivalent Intel FPGA resources

Xilinx Virtex UltraScale+	ALMs	REGs
ME kernel logic (estimated)	11 308.5 (1.31%)	25 250 (0.73%)
Available	864 000	3 456 000

**Table 5:** Estimated Arria 10 resource utilization for the kernel that works with extended frames, as reported by Intel’s compiler (*aoc*)

	ALMs	REGs	MLABs	RAMs	DSPs
Whole system(estimated)	$109\,233$ (25%)	$410\,662$ (24%)	$1\,448$ (2%)	766 (28%)	123 (8%)
Kernel syste (estimated)	$19\,258$ (5%)	$52\,090$ (3%)	$1\,448$ (2%)	274 (10%)	0 (0%)
ME kernel logic(estimated)	$17\,818$ (4%)	$48\,831$ (3%)	$1\,448$ (2%)	211 (10%)	0 (0%)
Available	$427\,200$	$1\,708\,800$	$42\,720$	$2\,713$	$1\,518$

To further analyze the effects OpenCL has over the resource utilization of a given FPGA design, we provide on Table 5 the resource utilization estimates for one of the OpenCL kernels when compiled for an Arria 10 FPGA. Only the resource estimates for the kernel version that works with extended frames are provided, since the VHDL implementation more closely resembles that particular version.

The first noticeable difference is that the VHDL kernels only use ALM and REG resources. When comparing the resource utilization results for Arria 10 FPGAs (Tables 3 and 5), we can see that using OpenCL generates a design that uses $1.35\times$ the ALMs and $1.94\times$ the REGs of the VHDL implementation. These, in terms of total FPGA resources, account for 1% more ALMs and 1.5% more REGs. In the case of the resource utilization results for data center FPGAs (Tables 2 and 4), for a fair comparison we will only use the compiler estimated resources for the Stratix 10 FPGA, and not the real ones. We can see that using OpenCL generates a design that uses $1.85\times$ the ALMs and $2.87\times$ the REGs of the VHDL implementation. These, in terms of total Stratix 10 resources, account for 1% more ALMs and 1.26% more REGs. It is worth noting that the VHDL design uses a systolic array to implement the macroblock and search area buffers and the OpenCL kernel does not. This discrepancy might account for part of the reduction in the amount of REGs utilized by the VHDL design. However, we consider this comparison to be fair, as we tried to develop an equivalent OpenCL kernel that used a systolic array, but the compiler was unable to infer the systolic array in a similar manner to the VHDL design.

### Performance

#### Experimental study against a CPU reference

The compilation reports generated by Intel’s compiler do not provide an estimate for the kernel’s latency, as other HLS frameworks do. Thus, we cannot provide a reliable evaluation of our proposal’s performance from a theoretical-only point of view. It is necessary to execute the kernels to measure their performance.

Before executing the different kernel versions, however, it is still useful to analyze and compare them using their respective working frequencies. Table 6 shows the working frequencies of our Full Search kernels. The kernel version that adds logic to detect border macroblocks has a lower working frequency. This might result in a lower performance compared to the other kernel, even though it computes fewer candidates per macroblock on average. Nevertheless, the working frequency alone is not enough to assert such hypothesis.

**Table 6:** Working frequency for the versions of Block Matching Motion Estimation using Full Search, as developed by Intel’s compiler (*aoc*)

	Frequency
Version with additional logic for border detection	308.00 MHz
Version that works with extended frames	316.00 MHz

An experimental study has been conducted to test the performance of our proposals, including both Full Search kernels and the preliminary Diamond Search kernel. A reference CPU implementation has been developed to make performance comparisons. This implementation is compiled with varying levels of optimization and vectorization capabilities. The experimentation consists in repeatedly performing Motion Estimations over two luminance-only Full HD frames (8 160 macroblocks each, 16 320 different macroblocks in total). Both frames are read from a file as raw, one-dimensional arrays of bytes, and sent to the FPGA using OpenCL API. Each iteration, the current and reference frames are swapped. The execution time for $1\,000$ iterations was noted. The experimentation was conducted in the Intel DevCloud platform, which comprises nodes with Stratix 10 FPGAs, and Intel Xeon Platinum 8256 CPUs. The platform’s CPUs operate at 3.80 GHz.

The different implementations compared in our study are:

1. the CPU reference version, compiled with -O2 optimization level and no vectorization,
2. the CPU reference version, compiled with -O2 optimization level and MMX vectorization (8 byte vector registers),
3. the CPU reference version, compiled with -O2 optimization level and SSE vectorization (16 byte vector registers),
4. the CPU reference version, compiled with -O3 optimization level and SSE vectorization (16 byte vector registers),
5. the FPGA version that adds border detection logic,
6. the FPGA version that works with extended frames, and
7. the preliminary Diamond Search FPGA version.

All the experimental scenarios involving an FPGA device were executed both in a CPU using the emulation mode, and in a real Stratix 10 FPGA.

The experimentation results are shown in Table 7. For Full Search, it can be seen that the version that works with extended frames achieves a slightly higher performance than the version that adds border detection logic. Both achieve a performance similar to that of the CPU reference compiled with -O3 optimization level. However, the energy consumption of the FPGA implementations is expected to be significantly lower. This expectation is supported by the fact that the working frequency of the CPU (3.80 GHz) is more than 12 times higher than that of the FPGA designs (308 and 316 MHz).

**Table 7:** Performance comparison between the FPGA kernels developed and the CPU reference compiled with varying levels of optimization

Version	Milliseconds/frame		Frames per second (fps)	
**CPU**				
Sequential reference (–O2)	1627.39		0.614	
–O2 + MMX	145.31		6.882	
–O2 + SSE	126.64		7.896	
–O3 (autovectorized with SSE)	89.49		11.174	
**FPGA**				
	Emulation(CPU)	Real FPGA	Emulation (CPU)	Real FPGA
Full search—With logic for border detection	1931.10	90.12	0.518	11.096
Full search—With extended frames	1796.92	88.43	0.557	11.309
Diamond Search (preliminary)	70.38	77.34	14.209	12.930

For Diamond Search, however, the results differ from the expected ones. When compared to Full Search, a speedup of around $30\times$ would be expected, since the amount of candidate blocks computed has been reduced by around $30\times$ on average. However, a speedup of only approximately $1.15\times$ is obtained. Moreover, emulating the Diamond Search kernel achieves a higher performance than executing it on a real FPGA, which is unusual. If we compare only the times obtained in the emulation executions, the Diamond Search version achieves a speedup of $25.53\times$ with respect to the Full Search one. This is much closer to the expected $30\times$ speedup. Such unusual behavior might indicate that the memory accesses have become the bottleneck for the Diamond Search version, and that our preliminary kernel uses memory access patterns that are inefficient for FPGA architectures. Whether this is a problem derived from using OpenCL, or just from an unoptimized code, is yet to be determined.

#### Theoretical comparison against an HDL implementation using performance estimations

Experimentally comparing OpenCL kernels with their corresponding HDL implementations requires not only to develop the HDL version of the kernel, but also to implement the communication interface with the host (e.g., PCIe hardware protocol). The latter is an enormous task that would require an extensive knowledge of PCIe, the transceivers in the FPGA, and even licensing protected technology. Regarding a theoretical-only comparison, using Intel’s compiler we do not have access to kernel latency data similar to that HDL synthesis tools, as well as other HLS synthesis tools, usually provide.

Since we consider of much interest to provide comparisons with HDL implementations, what we have done is to compare the measured data for the OpenCL kernels with estimated execution times from theoretical metrics of the equivalent VHDL design. This comparison is suboptimal, but nonetheless it provides additional insight about the behavior of OpenCL compared to HDL implementations.

We can estimate the VHDL implementation’s execution time from its latency and frequency. As it is the case with OpenCL for Intel FPGAs, the frequency is reported by the VHDL synthesis tools used. The VHDL synthesis tools used only allow targeting certain FPGA devices for compilation and performance estimations. Among the available options, we chose to target Intel Arria 10 FPGAs (which are less powerful than Intel Stratix 10 FPGAs), and Xilinx Virtex UltraScale+ FPGAs (which are data center FPGAs of similar performance to Intel Stratix 10 FPGAs). The reported frequencies are shown in Table 8. Regarding the latency, we estimate it as follows. The implementation loads 4-byte words into the systolic array each clock cycle. Thus, it completely fills the systolic array in $(16 \cdot 16 + 46 \cdot 46)/4 = 616$ cycles. After that, it computes one macroblock candidate per clock cycle, taking a total of $31 \cdot 31 = 961$ cycles to compute all candidates for one macroblock. That means that each macroblock is processed in $616+961 = 1\,577$ cycles, and a whole Full HD frame in $8\,160 \cdot 1\,577 = 12\,868\,320$ cycles.

Table 9 shows the estimated performance the VHDL implementation would achieve when executed in the chosen FPGAs, assuming a memory throughput high enough that the architecture never stalls. As it can be seen, the VHDL design would theoretically achieve more that $2 \times$, on an Arria 10, and $3 \times$, on a Virtex UltraScale+, the performance of the equivalent OpenCL kernel on a Stratix 10. It is worth noting that Arria 10 FPGAs are current-generation less-powerful alternatives to Stratix 10 FPGAs, and it is expected that the execution of the VHDL design on a Stratix 10 would achieve even higher performances.

**Table 8:** Working frequency for a VHDL implementation of Block Matching Motion Estimation using Full Search, when synthesized for 2 different FPGA families

Target device	Frequency
Intel Arria 10	333 MHz
Xilinx Virtex UltraScale+	475 MHz

**Table 9:** Estimated performance for a VHDL implementation of Block Matching Motion Estimation using Full Search, when executed in 2 FPGA families

Target device	Estimated milliseconds / frame	Estimated frames per second (fps)
Intel Arria 10	38.64	25.878
Xilinx Virtex UltraScale+	27.09	36.912

If the VHDL design were executed in an FPGA with a working frequency of 316 MHz, the same achieved by the equivalent OpenCL kernel, we estimate it could achieve a performance of 40.72 milliseconds per frame, or 24.556 fps. This is slightly more than $2\times$ the performance noted for the OpenCL kernel. The difference in performance with the estimated VHDL design could be caused by the following factors:

- The OpenCL design presents a latency of around double that of the VHDL design,
- The memory throughput of a real FPGA is not high enough to execute the design described without stalling, or
- A combination of the two above points.

### Evaluation of OpenCL as a tool for developing FPGA solutions

The main aim of this work is to evaluate OpenCL as a tool for developing video processing applications targeting FPGAs. The multiple kernels developed, together with the development process, allow us to form an opinion on that regard.

We consider OpenCL to be an interesting option when developing HPC applications targeting FPGAs. It efficiently abstracts away low-level electronic details from the programmer, allowing users to write FPGA applications using a more familiar language. The main advantage OpenCL has over any other HLS language is its broad adoption in HPC environments.

We noted that using OpenCL incurs a resource and performance penalty over low-level HDL implementations. This is to be expected, however. We believe the performance achieved by the OpenCL kernels to still be comparable to that of optimized CPU implementations. We also believe the resource penalty to be of little impact in the overall design when targeting a data center FPGA, as we did. Consequently, we consider these drawbacks to be a fair trade-off for the reduction in development efforts enabled by OpenCL.

Nevertheless, we have detected key issues with OpenCL as a tool for developing FPGA applications. First of all, its low portability to and from other accelerators breaks OpenCL’s main philosophy. To accelerate the same application using an FPGA and another accelerator, the programmer would have to write two different kernels. In addition, OpenCL for Intel FPGAs poses other limitations to the programmer, such as requesting the use of the OpenCL standard v1.0.

We also consider the compilation reports not returning any estimate for the kernels’ latency, as other HLS frameworks do, to be an important drawback. In addition, some information that is provided seems to be inaccurate or misleading. For example, the information provided by the Schedule Viewer (Beta), which does not accurately depict a kernel’s global flow.

We have detected that OpenCL has problems synthesizing certain codes. Specifically, we were not able to synthesize a kernel version that used a systolic array to implement the current macroblock and search area, similarly to the VHDL implementation developed.

Overall, we consider OpenCL for Intel FPGAs to be a promising choice for programming HPC applications on FPGAs using HLS. Nevertheless, it still lacks maturity in certain aspects, and thus it is not yet an optimal working environment.

## Conclusions

In this work, we have researched the viability of using OpenCL for Intel FPGAs (Intel FPGA SDK for OpenCL) to implement Block Matching Motion Estimation algorithms for video encoding and compression tasks. To do so, we have developed two OpenCL kernels performing Block Matching using the Full Search method, and one preliminary kernel using Diamond Search. Our proposals work with Full HD frames ($1\,920 \times 1\,080$ pixels) completely inside the FPGA. The SAD operations are computed entirely in parallel, and the Full Search kernels achieve high performance thanks to an efficient exploitation of the FPGA’s internal memory by means of OpenCL’s local memory features.

Our proposals have been developed targeting Intel Stratix 10 FPGAs, which are appropriate for intensive computations. The resource utilization for these FPGAs is relatively low for all kernels, using less than 10% of the resources for the kernel logic, and around 25% for the entire system.

We have conducted an experimental study comparing our Full Search implementations with a CPU reference version, compiled with varying levels of optimization and vectorization. The experimental results show that our Full Search proposals achieve a performance similar to that of the CPU reference when compiled with -O3 and SSE vectorization. We have also compared one of our Full Search implementations with estimates from an equivalent low-level VHDL implementation, in terms of resource utilization and performance. We observe that using OpenCL incurs an overhead in the resource utilization for the kernel logic, estimated to be of less than 2% of the total FPGA resources. It also incurs a performance penalty, achieving the OpenCL kernel a performance of around 47% that which was ideally estimated for the VHDL design.

We conclude that using high-level languages produces architectures that are close to those implemented manually, with a potential overhead in terms of area that we assess to be affordable in HPC applications targeting data center FPGAs. The advantages of using high-level languages are shorter design cycles, being less error-prone and flexibility.

The future work includes analyzing the problems detected with the Diamond Search implementation and fixing them, as well as expanding the experimentation to make comparisons with GPU and hybrid OpenCL-HDL implementations.

## Data Availability

The source codes and compilation reports generated during the development of this work are freely available on the following repository: https://gitlab.com/mandeca/me_opencl.
